# In situ conservation—harnessing natural and human‐derived evolutionary forces to ensure future crop adaptation

**DOI:** 10.1111/eva.12521

**Published:** 2017-09-06

**Authors:** Mauricio R. Bellon, Ehsan Dulloo, Julie Sardos, Imke Thormann, Jeremy J. Burdon

**Affiliations:** ^1^ Comisión Nacional para el Conocimiento y Uso de la Biodiversidad (CONABIO) México City México; ^2^ Bioversity International Maccarese Italy; ^3^ Bioversity France Montpellier Cedex 5 France; ^4^ Commonwealth Scientific and Industrial Research Organisation Agriculture & Food (CSIRO) Canberra ACT Australia

**Keywords:** agriculture, conservation, crop wild relatives, landraces, plant genetic resources

## Abstract

Ensuring the availability of the broadest possible germplasm base for agriculture in the face of increasingly uncertain and variable patterns of biotic and abiotic change is fundamental for the world's future food supply. While ex situ conservation plays a major role in the conservation and availability of crop germplasm, it may be insufficient to ensure this. In situ conservation aims to maintain target species and the collective genotypes they represent under evolution. A major rationale for this view is based on the likelihood that continued exposure to changing selective forces will generate and favor new genetic variation and an increased likelihood that rare alleles that may be of value to future agriculture are maintained. However, the evidence that underpins this key rationale remains fragmented and has not been examined systematically, thereby decreasing the perceived value and support for in situ conservation for agriculture and food systems and limiting the conservation options available. This study reviews evidence regarding the likelihood and rate of evolutionary change in both biotic and abiotic traits for crops and their wild relatives, placing these processes in a realistic context in which smallholder farming operates and crop wild relatives continue to exist. It identifies areas of research that would contribute to a deeper understanding of these processes as the basis for making them more useful for future crop adaptation.

## 1 INTRODUCTION

Protection and maintenance of the world's agricultural germplasm resources has never been more vital. The collection, maintenance, and classification of genetic resources of plants used in agriculture and forestry are vital processes underpinning the steady improvement of crop yields and humankind's ability to feed, clothe, and house an ever‐increasing global population. In response to these needs, protection of germplasm resources has received more or less international attention for the better part of a century (since Vavilov's pioneering work, see Vavilov, 1992), but current changes in global climate patterns with their significant regional implications have greatly enhanced concerns about the adequacy of protection measures (Food and Agriculture Organization of the United Nations [FAO], 2010, 2012; Intergovernmental Panel Climate Change [IPCC], 2014; Parmesan & Yohe, 2003). In this continuing challenge, ex situ collections play a major role in providing a readily available source of germplasm for the plant breeding community and in preserving geographically variable sources of genetic variation that might otherwise have been lost due to habitat loss. Furthermore, studies of individual and population samples of landraces and wild relatives deposited in ex situ collections have been an important source of knowledge regarding patterns of adaptation within species to a range of climatic, edaphic, and biotic factors (Franks, Sim, & Weis, 2007; Nevo et al., 2012; Thormann et al., 2017b; Thormann, Reeves, et al., 2017). Such associations have been used for predictive characterization of germplasm (Bari et al., 2014; Thormann et al., 2014; Thormann, Parra‐Quijano, et al., 2016) and to guide the gathering of additional germplasm.

Despite their undoubted value, ex situ collections have a fundamental limitation in that they are “frozen snapshots” reflecting the structure and genetic variation in individual populations at the time of collection (Brush, 2004; De Haan, Nuñez, Bonierbale, Ghislain, & van der Maesen, 2013; Wang et al., 2017). Once assembled, the alleles collected are fixed, and if not curated sufficiently well, will decline through genetic drift due to inappropriate regeneration procedures during storage (Gale & Lawrence, 1984; Harrington, 1972). In contrast, in situ conservation aims to maintain target species and the collective genotypes they represent growing within their natural environment (Brush, 2004). A major rationale for in situ conservation is to allow for the continuing evolution of target species in the face of changing selection pressures both naturally occurring and farmer‐induced that reflect altered agronomic practices, human preferences, and uses (Brush, 2004; Gepts, 2006; Vigouroux, Barnaud, Scarcelli, & Thuillet, 2011). This rationale is based on the likelihood of two components—(i) that continued exposure to changing selective forces will generate and favor new genetic variation and (ii) that existing rare alleles that may be of value to future agriculture are maintained (Bellon, 2009). Here, outcomes may be influenced by a wide range of factors including population size, generation time, the intensity of selection pressure, the genetic basis and heritability of the traits involved, the inherent plasticity of the species in question to abiotic stresses, and the extent to which local farming practices alter gene flow and selection through conscious retention of preferred variants.

However, the evidence that underpins this key rationale remains fragmented and has not been examined systematically, thereby decreasing the perceived value and support for in situ conservation for agriculture and food systems. In turn, this may lead to the maintenance of fewer options to sustain the genetic diversity needed to ensure crops can adapt in the face of global change. Furthermore, these evidentiary constraints limit the capacity to design and implement in situ conservation strategies and interventions that are practical in the real‐world contexts in which smallholder farming operates and crop wild relatives continue to exist. The aim of this study was to review basic premises regarding the likelihood and rate of evolutionary change in both biotic and abiotic traits for crops and their wild relatives, and particularly to place the former into a realistic context in which smallholder farming practices provide a dynamic, and potentially ever‐changing, overlay of human‐influenced selection pressures that can directly affect the evolution of new, or currently rare, genetic variation of value to the future of agriculture.

## 2 EVOLUTION IN TRAITS UNDER BIOTIC SELECTION PRESSURE

In both natural and agricultural settings, microevolutionary change in the relative frequency of disease resistance alleles already present within individual populations can occur over just a few years (Burdon, Groves, & Cullen, 1981; Ibrahim & Barrett, 1991; Meyers, Kaushik, & Nandety, 2005; Thrall et al., 2012; Webster, Saghai‐Maroof, & Allard, 1986). Such studies demonstrate the evolutionary pressures imposed by pathogens, underlining the importance of rare, preexisting resistance alleles as host populations change and diversify in the face of selection (cf. Red Queen dynamics; Hamilton, 1980). Theoretically, while a sufficiently large sample of individuals might be made as to capture all the extant genetic variation in a population, such samples would have to be improbably large. In contrast, given sufficient time and selection pressure, in real‐world populations, alleles that at one point in time were extremely rare may increase in frequency to the point at which they would be easily gathered in a subsequent sample (Frankham, Ballou, & Briscoe, 2010).

While microevolutionary changes are extremely important in the structuring and short‐term response of plant populations to selective pressures, from the viewpoint of justifying continuing efforts in in situ conservation, the more relevant question is how do novel resistance specificities at existing loci arise, and at what rate? Indeed, to date no studies have been reported that unequivocally demonstrate the de novo appearance of truly novel resistance alleles.

### 2.1 Qualitative (gene‐for‐gene) resistance genes

The use of a range of molecular technologies and extensive sequencing of genes in a wide range of plant species has generated a picture of five different classes of gene‐for‐gene resistance (R) genes, the majority of which are characterized by a consistent nucleotide‐binding site leucine‐rich repeat (NBS‐LRR) motif (Dangl & Jones, 2001; Meyer, Nelson, Clement, & Ramakrishnan, 2010). The generation of polymorphism in these resistance genes involves gene duplication, followed by DNA‐sequence divergence by point mutation, deletion, or duplication of intragenic DNA repeats. This variation is further diversified by reassortment between related genes (Ellis, Dodds, & Pryor, 2000). To directly address the question of whether reassortment can generate novel resistance specificities, Richter, Pryor, Bennetzen, and Hulbert (1995) screened 176 genetic recombination events within the Rp1 locus in maize (*Zea mays* subsp *mays*). Most events (>95%) showed no change in specificity; of the remainder, only four events were explained by the appearance of unaccountable novel specificities. The occurrence of these novel specificities aligns well with extensive occurrence of resistance gene analogs in a diversity of plants (Li et al., 2010; Quirin et al., 2012) and suggests that similar events are likely to arise on a continuing basis, albeit at low frequency, in most plant populations.

NBS‐LRR and other gene‐for‐gene type resistances are complex structures that are unlikely to evolve de novo again. However, there are a few documented examples where the same resistance gene confers protection to different pests (e.g., the tomato Mi‐1.2 gene confers resistance to root knot nematode, aphid, and whitefly in tomato: Nombela, Williamson, & Muniz, 2003). This raises the possibility that at any existing R gene, novel resistance specificities affecting previously untargeted pathogens may evolve through changes within those genes. This evolution could only occur in in situ situations. It is pertinent to note here that allelic series of different resistance specificities are commonly found in cultivated plant species e.g., wheat, maize, tomato, flax: (Chávez‐Medina, Leyva‐López, & Pataky, 2007; Hulbert, Webb, Smith, & Sun, 2001; McIntosh, Wellings, & Park, 1995) and evidence to date suggests that unequal crossing over during recombination is a major mechanism in generating such diversity (Hulbert et al., 2001; Zhu, Bennetzen, & Smith, 2013).

### 2.2 Adult plant resistance

While still controlled by the action of single genes, the structure and function of APR genes in cereals is quite different to qualitative resistance genes. APR genes are also effective against multiple pathogens (Lagudah et al., 2009; Mago et al., 2011) with only two to three base pair changes differentiating the resistant and susceptible alleles (Krattinger, Lagudah, Spielmeyer, Singh, & Huerta‐Espino, 2009). The small changes occurring between the susceptible and resistant alleles raise the interesting possibility that novel APR genes may arise within populations conserved in situ.

### 2.3 Quantitative resistance genes

Resistance controlled by the action of many genes each of different but relatively small phenotypic effect is a particularly common feature of plants attacked by necrotrophic fungi that kill host tissue. This resistance is associated with factors that may reduce infection rates (e.g., hairy leaves, few stomata) or slow the rate of spread within the plant (e.g., thicker cell walls, phenolic concentrations). Because many of these traits are continuous in their response, small changes in resistance are often difficult to detect (Burdon, 1987; Burdon, Barrett, Rebetzke, & Thrall, 2014). Furthermore, changes in resistance in response to pathogen attack may be correlated across multiple pathogen species (Mitchell‐Olds, James, Palmer, & Williams, 1995).

### 2.4 Observed changes in resistance of landrace populations

There are very few studies that provide direct evidence of temporal change in the resistance structure of wild populations even though this may occur with surprising rapidity (less than 6 years in *Linum marginale* L.; Thrall et al., 2012). Studies that compare the genetic structure of a recent sample with one from the same area that was deposited in an ex situ collection some time before are fraught with major problems of interpretation given the potential for temporally separated collections to target spatially close but separate populations (Jensen, Dreiseitl, Sadiki, & Schoen, 2011), and the inability to control for changes that may have occurred during storage (Parzies, Spoor, & Ennos, 2000).

## 3 EVOLUTION IN TRAITS UNDER ABIOTIC SELECTION PRESSURE

Physiological traits associated with nutrient uptake, response to cold, heat, and water stress tend to be controlled by the action of multiple genes (quantitative trait loci: QTLs) each of small phenotypic effect and in which genotype‐by‐environment effects are often very strong (Des Marais, Hernandez, & Juenger, 2013; Lowry et al., 2013). The genetic architecture of such traits—how variation is distributed in the genome; the extent of pleiotropic effects, and of plasticity—plays an important role in determining evolutionary responses to complex abiotic stresses (Alonso‐Blanco & Mendez‐Vigo, 2014; Clauw et al., 2016; Juenger, 2013). Because of the importance of developing co‐adapted gene complexes, the rate of evolutionary response to selection on many physiological traits is likely to occur at a slower rate to those controlled by genes with major phenotypic effect. However, because polygenic traits tend to evolve by subtle changes in gene frequency at many loci (Anderson, Willis, & Mitchell‐Olds, 2011), the potential for change is usually readily available. In this respect, the plasticity of individual genotypes will be of particular importance in the overall evolutionary response of populations to changing environments. Gradual changes in the environment are likely to be accommodated through plastic responses while abrupt changes will force more rapid selection (Nicotra et al., 2010). This may occur through recombination of existing QTLs, or through mutation including the formation of novel epialleles which can be triggered by various environmental stresses including drought (Golldack, Luking, & Yang, 2011; Shaik & Ramakrishna, 2012; Zhang, Fischer, Colot, & Bossdorf, 2013).

In contrast to selection for pest or disease resistance where the appearance of a new race or biotype may generate intense short‐term directional selection within individual populations, the greatest intensity and consistency of change in environmental variables tends to occur among populations across eco‐geographic clines. Adaptive differentiation as demonstrated by clinal patterns of response (e.g., to drying conditions: Shapter et al., 2012) attests to genetic changes by populations over broad geographic scales (Mercer & Perales, 2010). Examples of such broad‐scale adaptation are widespread including clines in freezing tolerance (Zuther, Schulz, Childs, & Hincha, 2012), seed traits influencing life cycle timing (Montensinos‐Navarro, Pico, & Tonsor, 2012), and flowering time (Keller, Levsen, Olson, & Tiffin, 2012). Notwithstanding this, even within individual populations, microenvironmental differences can sustain differential selection pressures leading to small‐scale spatial patterns and the maintenance of genetic variation responsive to abiotic factors (Nevo, Beiles, & Krugman, 1988; Verhoeven, Poorter, Nevo, & Biere, 2008).

Selection for phenological traits may occur very rapidly—with a number of studies showing responses in flowering time (Franks et al., 2007; Nevo et al., 2012). In a comprehensive study of the response of pearl millet (*Pennisetum glaucum* (L.)R.Br.) in the Sahel to recurrent drought over the last quarter of the 20th century, no major changes were detected in the main cultivated varieties. However, common garden comparisons of landraces collected at the same locations 27 years apart found significant shifts in adaptive traits—reductions in plant size, spike length, shorter life cycles, and an increase in the frequency of a flowering gene known to affect development (Vigouroux, Cedric, et al., 2011). In this situation, short‐term adaptation to climatic variation was driven through selection on existing variation in in situ populations—not through the adoption of new varieties. Again, this provides a powerful message about the importance of allowing continued evolution in the face of changing environmental conditions. In situ conservation maximizes the chances that rare alleles are potentially available to allow plants to adapt through the development of new combinations of existing variants; standard sampling strategies for ex situ conservation, on the other hand, will fail to capture such variation, thereby reducing future options.

## 4 ON‐FARM MANAGEMENT AND THE PRACTICALITIES OF IN SITU CONSERVATION

Traditionally, in situ conservation has included consideration of (i) landraces of mainstream agricultural crops, and underutilized and neglected crops, as well as (ii) wild crop relatives and forest tree resources. While these two categories have a number of issues in common, in reality there are also significant differences. In situ conservation of wild relatives and forest tree resources focuses on responding to the drivers and pressures that threaten the natural populations so as to maintain the genetic diversity and geographic range of species, thereby maximizing their potential to respond to natural or human‐made environmental change. In contrast, in situ conservation of landraces of mainstream agricultural crops and of underutilized and neglected crops represents a more complex selection environment where the impact of response to naturally occurring selective forces is overlain with conscious selection by farmers, with deliberate movement and incorporation of germplasm from close and more distant sources (including both more advanced cultivars and wild relatives), and with a range of cultural practices.

In the case of crops, a large amount of diversity is still retained in developing countries by smallholder farmers (Van de Wouw, Kik, van Hintum, van Treuren, & Visser, 2010), particularly for many crops in their centers of domestication and diversity. There, farmers continue to grow landraces and maintain traditional knowledge and seed management practices (Brush, 2004; Jarvis et al., 2008), a process known as de facto conservation (Brush, 2004). There is an increasing body of literature that documents how these farmers maintain and influence important amounts of phenotypic and genetic diversity of crops with different reproductive systems and evolutionary histories, for example, for maize in Mexico (Orozco‐Ramirez, Ross‐Ibarra, Santacruz‐Varela, & Brush, 2016; Perales, Benz, & Brush, 2005; Pressoir & Berthaud, 2004a,b), potatoes (*Solanum tuberosum* L.) in Peru (De Haan et al., 2013; Quiros et al., 1992), rice (*Oryza sativa* L.) in China (Wang et al., 2017), barley in Ethiopia (Samberg, Fishman, & Allendorf, 2013), sorghum (*Sorghum bicolor* (L.) Moench) in Cameroon (Barnaud, Deu, Garine, McKey, & Joly, 2007) and in Kenya (Labeyrie et al., 2014, 2016), pearl millet in Kenya (Labeyrie et al., 2016), and cassava (*Manihot esculenta* Crantz) in Guyana (Elias, McKey, Panaud, Anstett, & Robert, 2001) among others (see Supporting Information for some relevant results from these studies).

Farmer seed management is a strong determinant of spatial structure in crop genetic resources—a fact that highlights the importance of intermeshing social, landscape, and genetic data into the design of germplasm conservation strategies (Labeyrie et al., 2014, 2016; Orozco‐Ramirez et al., 2016; Samberg et al., 2013). Recognition of this human involvement and its significant effect on the structure of local and regional crop populations increases the need to recognize in situ conservation on‐farm of crop species as a dynamic evolutionary process (Barnaud et al., 2007; Labeyrie et al., 2014, 2016; Orozco‐Ramirez et al., 2016; Pressoir & Berthaud, 2004a,b; Samberg et al., 2013; Vigouroux, Barnaud, et al., 2011; Wang et al., 2017) It is distinctly different to that occurring in wild relatives and noncrop species where the homogenizing effects of seed exchange/sharing and the accelerated selective forces of rogueing of susceptible individuals are not imposed.

The justification for in situ conservation on‐farm depends on the existence of crop evolution under farmer management. To date, the most comprehensive experimental evidence of crop microevolution we are aware of has been gathered for bread wheat (*Triticum aestivum* L.) by scientists at the French National Institute for Agricultural Research (INRA) under an approach they call dynamic management (DM) of crop diversity. For a review summarizing their results see Enjalbert et al. (2011), key relevant findings are presented below. The approach consisted of planting composite diverse wheat populations under a range of environmental conditions across France and letting them evolve while monitoring the process. While the approach did not involve farmers per se (comprising INRA research stations and agricultural high schools) and was carried out in a developed country, it is quite relevant to in situ conservation on‐farm as they were able to measure specific results of evolution in crop populations for over 26 years. Results show increases in plant height, rapid evolution in earliness traits, and divergent selection on flowering time responding to climatic conditions. In particular, for the latter, in two of three environments studied, different allelic combinations were selected and the emergence of new alleles that were not detected in parental lines was identified. They concluded, however, that to maintain crop populations with good agronomic value—thus useful for humans—require the involvement of human selection for some key traits. They also report on how networks of farmers have been involved in efforts that build on the DM approach to generate varieties suitable for organic farming and low‐input agriculture. They show, for example, that the diversity conserved on farm is not a duplicate to that conserved in the gene bank, the diversity of the former was greater than that of the latter, and alleles present on farm were different from those in gene bank accessions.

Landraces and the farmers who maintain them essentially constitute “coevolving” sociobiological systems (Bellon, Gotor, & Caracciolo, 2015a). For any given crop, farmers influence through their knowledge, preferences and practices, the alleles and genotypes that pass from one crop generation to the next (Bellon, 2009; Gepts, 2006; Vigouroux, Barnaud, et al., 2011). Traditional practices of saving and sharing seed in network structures that connect farmers and landraces within and across environments underpin these sociobiological systems and are an essential component to understand the spatial structure of crop genetic resources and their dynamics (Labeyrie et al., 2014, 2016; Pautasso et al., 2013; Samberg et al., 2013).

These systems depend crucially on farmers' incentives, institutions, and social organization (Bellon, 2004; Brush, 2004; Negri, 2003; Zimmerer, 2010). De facto conservation continues because the farmers involved obtain direct benefits from the diverse landraces they grow, such as (i) optimizing crop production under agroecological heterogeneous conditions, particularly in marginal areas; (ii) managing risk; (iii) producing a variety of products with different uses; (iv) profiting from commercial opportunities in niche markets; (v) providing themselves with appreciated varieties due to consumption qualities or cultural significance; (vi) managing labor during the agricultural season (Bellon et al., 2015a) and thus have incentives to maintain them. In fact, de facto on‐farm conservation may be the only way some farmer communities manage to obtain benefits from many crops that are important to them but neglected by formal research or commercial entities or under conditions where there is no institutional support to address their needs (Padulosi, Heywood, Hunter, & Jarvis, 2011). The challenge however is that many of these farmers increasingly face strong incentives to abandon their landraces and the processes that sustain them due to social, economic, environmental, and cultural changes (Bellon, 2004; Negri, 2003; Van de Wouw et al., 2010; Zimmerer, 2010). These drivers are complex (Bellon, 2004; Brush, 2004; Van de Wouw et al., 2010). As summarized by Bellon et al. (2015a), specific reasons to abandon crop diversity include the following: (i) availability of scientifically bred varieties with higher yields and better disease resistance, that, together with of the use of external inputs such as fertilizers, may foster specialization and the replacement of a broad array of local varieties for just a few; (ii) development and increasing reach of modern value chains that may make traditional value chains linked to niche markets uncompetitive, leading to fewer commercial opportunities for marketing diverse varieties or products derived from them; (iii) availability of new products may compete with products derived from traditional crops or local varieties in terms of price and convenience, which together with changes in taste, or an increased perception that traditional crops and varieties are associated with poverty or low social status, may reduce their appeal; (iv) increased migration and off‐farm labor opportunities can decrease the feasibility of maintaining crop diversity on‐farm, by decreasing labor supply and increasing its opportunity cost. Indeed, migration and off‐farm labor opportunities also can provide alternative sources of income to manage risk, thereby reducing the need to maintain crop diversity. In particular, increased availability of formal seed systems may lead farmers to abandon traditional seed management practices such as seed saving, selection, and sharing in favor of purchasing seed and through this, stopping processes of crop evolution (Vigouroux, Barnaud, et al., 2011). Furthermore, there is increasing evidence that farmers see value in incorporating improved varieties into their systems where they are subject to the same evolutionary processes as landraces, also known as “creolization” (Bellon, Adato, Becerril, & Mindek, 2006; Westengen, Ring, Berg, & Brysting, 2014). Supporting in situ conservation on‐farm in these sociobiological systems may increasingly require outside intervention to ensure that incentives are sufficiently attractive to farmers (Bellon, 2004; Jarvis, Hodgkin, Sthapit, Fadda, & Lopez‐Noriega, 2011; Narloch, Drucker, & Pascual, 2011).

In the last 20 years, many projects have been implemented worldwide to support on‐farm conservation of different crops. An extensive review (Jarvis et al., 2011) identified 59 different types of interventions for supporting on‐farm conservation worldwide, but there has been little empirical evidence that they actually made a difference beyond what de facto conservation already achieves. Efforts have tended to be ad hoc, small scale, fragmented, and uncertain in terms of their impact (Bellon et al., 2015a). There is, however, some recent systematic evidence that interventions implemented to support on‐farm conservation can lead to higher levels of phenotypic diversity and livelihood benefits than would have been possible without them for Andean crops (Bellon, Gotor, & Caracciolo, 2015b) and for phenotypic diversity only in the case of fruit trees in Central Asia (Gotor et al., 2017). To our knowledge, there is still a lack of evidence that interventions associated with on‐farm conservation projects lead to additional outcomes related to genetic diversity and crop evolution—an area that merits further research.

The potential value of the genetic variation under evolution for use in other regions, under different circumstances, or changing conditions is fundamental because it is this value to broader society that justifies supporting specific sociobiological systems. A key question is how to harness this value? Our argument suggests that a guiding principle should be identifying rare or new variation associated with adaptive traits under changing or contrasting conditions and makes this variation available to other farmers, communities, breeders, or others where it can be useful. Implementing this principle requires strong collaboration among farmers, scientists, other social actors (e.g., extension workers, activists), and institutions (e.g., NGOs, local governments, schools), as well as more concerted and systematic efforts that build on the best available biological and social sciences. This may require the creation of mechanisms to monitor the status and trends of crop diversity, adaptation, and evolutionary processes, based on methodologies and mechanisms to target where and with whom to carry out the monitoring and how to identify useful variation (Caldu‐Primo, Mastretta‐Yanes, Wegier, & Piñero, 2017). Adapting methodologies such as predictive characterization that have been used to identify populations likely to contain specific traits and thus guide targeted collection and germplasm collection (see Thormann et al., 2014 for a review) could be used to guide and target the monitoring and recurrent sampling of locations where new useful genetic variation of a crop is likely to appear. Mechanisms should build on the knowledge and methodologies of studies on the structure, evolution, and adaptation of landraces under farmer management reviewed above (Mercer, Martínez‐Vásquez, & Perales, 2008; Pressoir & Berthaud, 2004a,b; Vigouroux, Cedric, et al., 2011), as well as take into consideration the broader social and ecological landscapes where diverse landraces are maintained by different farming communities (Labeyrie et al., 2014, 2016; Samberg et al., 2013). The institutional, scientific, and physical infrastructure that has been developed as part of ex situ conservation can be also an asset for these efforts. Gene banks are more than repositories of seeds—they contain a great deal of information about diversity (genetic, geographic, phenotypic, etc.), and very importantly, experience on how to access and monitor crop diversity at national and global levels. For example, comparison of accessions from gene banks to samples collected periodically from farmers could provide a means of assessing genetic changes (see Section 3 below).

Monitoring efforts should not only focus on genetic variation, but also assess the incentives that farmers have to maintain crop evolution in their fields. In many circumstances, farmers may continue to have sufficient internal incentive as to preclude the need for outside intervention. In other situations, however, interventions may be needed. Interventions should be well‐targeted and build on the knowledge and evidence we have about the socioeconomic and cultural factors that favor or hinder maintaining crop diversity on farm. They should involve mechanisms to assess whether these interventions are effective or not (see Bellon et al., 2015a for a framework to assess interventions from a livelihoods perspective).

It is important to emphasize that the value of novel or rare genetic variation should not be seen only through the lens of its use in formal breeding efforts; rather, it is crucial to recognize its direct benefit to farmers (Perales, 2016): for example, the identification and sharing of “interesting” landraces among farmers in different locations (Bellon et al., 2003), the integration of this variation into participatory plant breeding efforts with local communities (Cecarrelli, Grando, & Baum, 2007), or through evolutionary breeding efforts (Murphy, Bazile, Kellogg, & Rahmanian, 2016; Perales, 2016; Raggi et al., 2017).

An important consideration for the contribution of on‐farm conservation to the enhancement of the capacity of crops to adapt to novel future conditions is to recognize that evolution is a “numbers game.” It is not enough just to have a few farmers or communities maintaining crop diversity and associated practices; rather successful on‐farm conservation needs continuing commitment by numerous farmers and communities to participate in the process. For example, in Mexico, the center of origin and a center for diversity of maize (Doebley, 2004; Hufford et al., 2012), about 2 million smallholder farmers (Fernandez Suarez, Morales Chavez, & Galvez Mariscal, 2013), planted around 4.7 million hectares under rainfed conditions in 2010 (Table S1), most of them relying on traditional practices of saving and sharing seed. If one assumes a planting rate of 30,000 plants/ha (Mercer et al., 2008), this means that circa 141 billion maize plants growing across 11 distinct biogeographic regions (Perales & Golicher, 2014) are subject to on‐farm evolutionary pressures every year. As a consequence, the probability that mutations appear, or rare alleles are maintained, that could be adaptive in the future is substantial. Assessing these numbers is beyond the scope of this paper but an important task for the future.

Creating and sustaining mechanisms that build on the experience and knowledge of farmers to support and monitor crop evolution on farm and make its outcomes available to other users face multiple challenges. A major risk inherent in these sociobiological systems is their dependence on the decisions of many households who may decide not to continue to be involved (Brush, 2004). At the same time though, this is also a strength as it increases the probability that at least some participants will remain involved in the long run. There are important policy barriers that may limit the viability of these mechanisms, particularly increasing local and global restrictions on access to seeds and germplasm (Gepts, 2006; Halewood, 2013; Louafi, Bazile, & Noyer, 2013; Louafi & Schloen, 2013). Local constraints often reflect national policies that favor the recognition of uniform, scientifically bred varieties over more heterogeneous, variable landraces; global constraints result from countries asserting sovereignty over plant genetic resources found within their national boundaries (Halewood, 2013; Louafi & Schloen, 2013; Moore & Hawtin, 2014). The belief that significant monetary benefits can be gained from “sovereign” seed (genetic resources over which a native community has controlling rights) can encourage restriction of access and contribute to reductions in the global flow of plant genetic resources (Falcon & Fowler, 2002; Louafi & Schloen, 2013). Furthermore, issues of obtaining prior informed consent to collect and share material and benefit sharing mechanisms are important considerations that have to be taken into account to insure that the benefits from evolutionary processes are shared equitably (Louafi & Schloen, 2013).

On‐farm conservation as a strategy for conserving and using plant genetic resources is then about maintaining dynamic sociobiological systems as sources of currently rare or new genetic variation of value to the future of agriculture. It builds on farmers' knowledge, practices, incentives, and the crop populations they manage, recognizing these farmers as key actors in the process. Maintaining these systems must be compatible with improved livelihoods and well‐being for them while simultaneously creating equitable mechanisms that allow society at large to access this novel variation to face the challenges posed by ever‐changing environments.

## 5 IN SITU CONSERVATION OF CROP WILD RELATIVES

Crop wild relatives (CWR) are wild species living and evolving in natural, semi‐wild, and/or human‐made habitats where their genetic diversity is affected by a wide range of factors including habitat fragmentation and degradation (Millennium Ecosystem Assessment [MEA], 2005). Their genetic relationship with cultivated land races is summarized in the concept of primary, secondary, and tertiary genepools (Harlan & de Wet, 1971). Wild relatives that are part of the primary, secondary, and tertiary genepool of a crop potentially can continue to contribute to ongoing genetic change in the crop variety; however, depending on the genepool level, the ease with which genes can be transferred to crops is progressively more difficult (Harlan & de Wet, 1971; Maxted, Ford‐Lloyd, Jury, Kell, & Scholten, 2006).

Losses in intraspecific genetic variation within populations affect their ability to respond to evolutionary pressures engendered by environmental and climatic change and may result in reduced fitness, loss of ecosystem functioning, and recovery (Reusch, Ehlers, Hammerli, & Worm, 2005; Whitham et al., 2003). Ultimately, this may jeopardize population persistence (Spielman, Brook, Briscoe, & Frankham, 2004) as well as the species richness of plant communities (Booth & Grime, 2003). In situ conservation of CWR is often limited to species occurring in protected areas established with other reasons in mind (Dulloo et al., 1998; Maxted, Dulloo, & Eastwood, 1999). Few reserves have been established with the specific purpose of CWR conservation, but see, for example, for wheat relatives in Armenia (Avagyan, 2008) and Israel (Anikster, Feldman, & Horovitz, 1997), and teosinte (*Z. diploperennis*) in southwest Mexico (United Nations Educational, Scientific, and Cultural Organization [UNESCO], 2007). As a consequence, where CWR populations occur in protected areas, they are largely conserved passively (Maxted & Kell, 2009) and are thus still threatened by invasive species, habitat degradation, and untargeted management (Hunter & Heywood, 2011).

With limited resources available for conservation, the challenge for in situ conservation of CWR is to first prioritize species and the number of populations that would conserve the maximum genetic diversity (Dulloo et al., 2008; Magos Brehm, Maxted, Ford‐Lloyd, & Martins‐Loução, 2008; Maxted, Ford‐Lloyd, & Hawkes, 1997). Many different genetic approaches based on evolutionary isolation and phylogenetic relatedness have been proposed for prioritizing species and populations. For example, Weitzman (1992) used expected diversity to identify the set of taxa that would retain the most diversity on a future phylogenetic tree, given some measure of diversity and a probability of persistence for each potential combination of taxa. Bonin, Nicole, Pompanon, Miaud, and Taberlet (2007) also showed that the principle of complementarity deserved to be used more often. Importantly, they argued the need to focus on adaptive traits within wild species and developed a new index that takes account of the adaptive value of populations. Furthermore, they demonstrated that using more traditional neutral markers as opposed to adaptive methods resulted in different populations being selected for protection. In practice, the principle of complementarity is used in designing genetic reserves to make the optimal use of available resources and maximize the number of protected species (Cabeza & Moilanen, 2001; Margules & Pressey, 2000). Other predictive characterization methods, including Focused Identification of Germplasm Strategies (FIGS; Street et al., 2008) and the ecogeographical filtering method (Thormann et al., 2014), have been used to identify adaptive abiotic and biotic traits in wild populations of CWRs.

## 6 WILD CONTRIBUTIONS TO POSTDOMESTICATION IN SITU DIVERSIFICATION

In the past, domestication has typically been associated with marked genetic bottlenecks as one or a limited number of events are involved, and subsequent conscious or unconscious selection by farmers leads to a further narrowing of the gene pool. However, the widespread use of marker technologies has led to a revision of this view with domestication now seen as a continuum of ongoing processes, involving the initial extraction of plants from their wild habitats and subsequent further diversification events (Gepts, 2004; Shigeta, 1996). The contribution of wild relatives to this secondary diversification of crops is receiving increasing attention and includes repeated episodes of temporally separated introgression from wild relatives into apple (*Malus pumila* Miller, 1768; Cornille et al., 2012), almond (*Prunus dulcis* (Mill.) D. A. Webb; Delplancke et al., 2011), and maize (Hufford et al., 2013). These studies, among others, revealed that secondary introgression of wild genepools into crop species has significantly contributed to shaping current crop genetic diversity although the extent of this varies among species. In barley (*Hordeum vulgare* subsp. *vulgare*), significantly higher levels of diversity are encountered in wild, compared to cultivated forms (Russel et al., 2004, 2011). In contrast, gene flow between cultivated carrot (*Daucus carota* subsp. *sativus*) and its wild relatives has been so intense that there is no evidence of a genetic bottleneck in the cultivated form (Iorrizo et al., 2013). It is highly likely that this continuing process of wild plant–crop introgression contributes to crop adaptation to specific conditions in many species.

Domestication and introgression events affecting food resources are not the sole preserve of the past. Particularly in traditional, subsistence‐oriented, agroecosystems, ongoing evolutionary processes involving wild relatives of mainly “minor” crops have been documented. In Ethiopia, despite the vegetative mode of propagation of Ensete (*Ensete ventricosum* (Welw.) Cheesman), gene flow from the wild population to the crop occurs through the regular incorporation of seedlings within cultivated plots (Shigeta, 1996). In Benin, another vegetatively propagated crop, yam (*Dioscorea cayenensis* subsp. *rotundata* (Poir) J. Miege) is regularly re‐domesticated as farmers collect, test, and select plants from neighboring natural populations (Chaïr et al., 2010; Scarcelli et al., 2006), while the columnar cactus *Stenocereus pruinosus* (Otto ex Pfeiff.) Buxb. is also undergoing frequent wild‐to‐crop introgression through the regular incorporation of cuttings collected in the wild (Parra et al., 2010). These examples highlight continuing interaction between wild crop relatives and their domesticated brethren, and the importance of the former in influencing on‐farm evolution of cultivated crops. In these interactions, local farmers play a vital role.

## 7 COMPLEMENTARITY BETWEEN EX SITU AND IN SITU CONSERVATION

Ex situ and in situ conservation are today considered as complementary conservation strategies, as both have specific advantages and disadvantages, and neither is sufficient in themselves to conserve the existing and evolving diversity of a species (Dulloo, Rao, Engelmann, & Engels, 2005; Gepts, 2006; Hunter & Heywood, 2011; Maxted et al., 1997). The final choice of specific in situ and ex situ conservation actions depends on the following: (i) considering the species biology and its performance under storage; and (ii) the intended use of the germplasm being conserved. Crop wild relatives, whose main value is considered to be the provision of adaptive genetic diversity for plant improvement, are preferably conserved in situ as this allows further evolution to occur (Maxted et al., 1997). However, in some cases, exposure to the natural environment constitutes a threat to the survival of part or all of the diversity of a species (e.g., due to habitat destruction). In these cases, complementary ex situ conservation can contribute to achieving optimal and safe conservation of the species' genetic diversity. Germplasm stored ex situ can also support in situ conservation efforts by providing a source of material for the reintroduction of species that have disappeared from their natural environment. Plant material from ex situ collections may also be used in enrichment plantings or reenforcement of threatened CWR populations and those which are not regenerating in the wild (Dulloo, 2011).

Ex situ conservation and in situ conservation are also complementary from an evolutionary research point of view. To understand evolutionary responses induced by biotic and abiotic pressures, ex situ collections potentially can be very useful sources from which to resurrect historical genotypes to compare with contemporary populations (Franks et al., 2008; Thormann, Fiorino, Halewood, & Engels, 2015). Large numbers of samples of threatened landraces and crop wild relatives collected in the past are stored in gene banks. Many of the collecting missions were sufficiently well documented as to allow precise identification of past collecting sites, thereby allowing sites to be revisited and populations, if still extant, recollected to compare with historical seeds (De Haan et al., 2013; Thormann & Dulloo, 2015; Thormann et al., 2017b; Thormann, Reeves, et al., 2017; Vigouroux, Cedric, et al., 2011; Wang et al., 2017). As noted earlier, historic and contemporary genotypes of wild cereals (Nevo et al., 2012) and of field mustard (Franks et al., 2007) sampled from the same locations showed evidence of advancement of flowering time due to climate change. While ex situ collections provide historic data and material for in situ monitoring of diversity and assessment of evolutionary changes, in turn, the results of such studies will inform and improve in situ conservation strategies. Ex situ conservation is a vital component of endeavors such as “Project baseline” (Franks et al., 2008), which is monitoring contemporary populations of a wide range of wild species in conservation sites and aims to regularly collect and store seeds from these populations in order to make available collections of time series samples for future evolutionary studies.

## 8 FUTURE RESEARCH ISSUES

It is vital that the genetic diversity underpinning the world's crops is protected and enhanced. There are multiple paths to achieving components of that aim. Here, we provide examples of research topics that would contribute to a deeper understanding of the processes whereby existing variation is maintained and new variation generated in wild reserves and farmer's fields and could be the basis for making these processes more useful:

Measuring the extent to which extant ex situ collections actually represent the genetic variation of the species in question present within distinct eco‐geographic regions, agroecological regions, and agricultural systems. [This would help focus the relative magnitude of future ex situ and in situ conservation efforts].

Assessing of the rate of change in host–pathogen diversity in smallholder agricultural settings and its impact on productivity. [This would provide a measure of the dynamism of individual systems and their responsiveness to management].

Determining the rate and importance of epigenetic change in generating novel variation in abiotic traits. [This would provide a measure of the likely adaptability of populations close to their current environmental limits to climate change].

Monitoring rate of change in genetic diversity and population dynamics over time in crop wild relatives [This would help prioritize populations and design in situ management actions].

Developing models that synthesize knowledge and evidence of population and landscape genetics in the context of farmers' practices, to explore the scale and scope of farmer involvement needed to maintain evolutionary processes likely to generate new, or currently rare, genetic variation of value to the future of agriculture [This will help design realistic interventions to support these processes].

Predictive characterization has been used to identify populations likely to contain specific traits and thus guide targeted collection and germplasm collection. Similar techniques are needed to guide and target the monitoring and recurrent sampling of locations where new useful genetic variation of a crop are likely to appear [This will help to make the process of evolution useful for agriculture].

## 9 CONCLUSIONS

A good measure of the effectiveness of in situ conservation of germplasm depends on evidence of continued evolution and diversity within and among populations, and on the use of these diversity and evolutionary outcomes beyond the situations where they take place. Clearly, a solid body of empirical evidence in support of this will take time to accumulate. However, circumstantial evidence provided by geographic‐scale patterns in diversity that correlate with major biotic and abiotic factors backed up by an increasing number of examples of short‐term evolutionary responses to pathogens and climate variability already provides strong support for the evolutionary rationale for in situ conservation.

Ex situ conservation of genetic resources is an extremely important endeavor providing security against loss of diversity in the field and ease of access, and hence usage, by plant improvement and breeding programs. However, even in the most extensively collected species, concern still exists as to the geographic and environmental representativeness of collections. While rapid advances in molecular technologies suggest that artificial evolution in some traits (e.g., some types of disease resistance) may become increasingly important, within‐species evolution of more complex traits (e.g., multigenic disease and pest resistance; drought tolerance) is still well beyond the horizon. For less well‐collected species such as many crop wild relatives and the large numbers of neglected and underutilized species that have little or no representation in ex situ collections, these concerns are magnified many times.

In situ conservation on‐farm remains a vital part of ensuring germplasm availability for use by future generations. Evolution in these highly important situations is determined by a complex of interactions between crop, environment, and humans at a range of spatial scales. Social factors involving the full gamut of interactions from relationships between adjacent and more far‐flung communities, to taste preferences and traditional beliefs, ensure that farmers and landraces constitute a complex coevolving sociobiological system. There is strong circumstantial evidence that even without the added human dimension, evolution can lead to the de novo appearance of novel alleles or the selection of favorable gene complexes that adapt plants to changes in their biotic and abiotic environments.

The added human component that is an integral part of in situ conservation on‐farm can drive evolution at an even faster pace through measures that lead to repeated introduction of additional genetic variation, while simultaneously enforcing tough selection pressures through active management of less desirable characteristics. Understanding the extent of this process and its impact on the genetic identity of landraces used in subsistence agriculture is a vital component in ensuring the maintenance of diversity into the future.
