# Supplementary material for: In situ conservation—harnessing natural and human‐derived evolutionary forces to ensure future crop adaptation
Source: Evol Appl. 2017 Sep 6;10(10):965–77. doi: 10.1111/eva.12521 (PMC5680627; doi:10.1111/eva.12521)
Supplement: Supplementary file 1 [file EVA-10-965-s001.docx]

Table S1. Area planted to maize and associated number of municipalities under rainfed conditions during the Spring-Summer cycle (May-October) of 2010 grouped by average yield-level at the municipal level in Mexico

| Yield level  (ton/ha) | Area planted  (ha) |  | Number of  municipalities |
| --- | --- | --- | --- |
| ≤ 1 | 1,851,591.0 |  | 878 |
| >1- ≤ 2 | 1,714,620.4 |  | 735 |
| >2- ≤ 3 | 1,120,841.5 |  | 384 |
| >3- ≤ 4 | 639,128.3 |  | 146 |
| >4- ≤ 5 | 230,026.2 |  | 57 |
| >5- ≤ 6 | 130,196.9 |  | 27 |
| >6 | 290,797.8 |  | 43 |
| Sub-total (≤ 1--3)* | 4,687,052.8 |  | 1997 |
| Total | 5,977,202.1 |  | 2270 |

Source: Sistema de Información Agroalimentaria y Pequera (SIACON) <http://infosiap.siap.gob.mx/aagricola_siap_gb/icultivo/>

*Yield range considered to be of smallholder maize farmers
