# Supplementary material for: In situ conservation—harnessing natural and human‐derived evolutionary forces to ensure future crop adaptation
Source: Evol Appl. 2017 Sep 6;10(10):965–77. doi: 10.1111/eva.12521 (PMC5680627; doi:10.1111/eva.12521)
Supplement: Supplementary file 2 [file EVA-10-965-s002.docx]

Supporting Information. Examples from studies documenting the role of farmers in maintaining and influencing phenotypic and genetic diversity of landraces under in situ conservation

Here we summarize some key results from some studies that have documented the role of farmers in maintaining and influencing phenotypic and genetic diversity in landraces they grow of different crops in diverse locations. The selected studies are not exhaustive and are only intended to provide the reader with examples of the results that have been obtained. The studies included present a combination of phenotypic and genetic data, with samples coming directly from farmers growing the crop in centers of diversity. The crops included have different breeding systems and propagation methods: allogamous and seed (maize, pearl millet), allogamous vegetatively propagated (potatoes and cassava); autogamous and seed (barley, sorghum, and rice) and include both propagation by seed (maize, pearl millet, barley, sorghum, and rice) and vegetative parts (tubers –potato, stem cuttings-cassava). Some of the studies also present a comparison between in situ and ex situ conservation.

**Maize in Mexico**

In a pioneering study of maize landraces collected from traditional famers, Pressoir & Berthaud (2004a,b) described the impact of farmers´ selection on population differentiation for quantitative traits and compare this to equivalent data based on molecular SSR markers. They carried out their study in three villages in southeastern Mexico where they found strong divergent selection among the villages for quantitative traits, but no spatial structuring for genetic markers reflecting considerable gene flow among the populations. They concluded that farmers´ decisions and selection strategies had a great impact on phenotypic diversification of landraces, but at the genetic level seed-mediated gene flow associated with the farmers´ seed selection practices and systems, tend to homogenize the maize landrace populations.

Perales, Benz & Brush (2005) studied 9 communities in the highlands of southeastern Mexico. These communities were from two different ethnic groups located in the same general region. They collected maize samples from farmers in these communities, as well as a farmer survey. The ears were characterized for morphological traits then planted in reciprocal common gardens to assess the agronomic performance of the samples in the different locations from which they were obtained. Isozymes were used to generate data for a genetic analysis. Morphological and agronomic differences occurred among the landraces planted by the two different ethnic groups, but there was small genetic distance and weak population differentiation among the locations. Each group maintained their own landraces in spite of reciprocal adaptation of the landraces of both groups to each other’s environment and superior yield performance of the landraces of one group in both environments. They concluded that ethnolinguistic identity plays a key role in maintaining landrace diversity.

Orozco-Ramirez et al. (2017) study maize landraces in southeastern Mexico obtained directly from farmers in villages at different elevations in two adjacent municipalities of differing ethnicity. They planted the sample in reciprocal common gardens and characterized their phenotypes, as well as carrying out genetic analysis using SSR markers. In contrast to the previous studies, they found modest morphological and genetic differentiation. Seed flows were found to be limited due to poor road infrastructure and most foreign seed was obtained from villages of the same ethnicity, even though distance between villages of different ethnicities can be shorter. In addition, an absence of common local or regional markets restricted the potential for seed exchange among different ethnic groups. These authors attributed their results to limited seed-mediate gene flow among villages due ethnolinguistic differences that isolated maize populations.

**Sorghum in Cameroon**

Bernaud et al. (2007) studied sorghum landraces in one village in northern Cameroon. They collected samples directly from farmers in the village, as well as using ethnographic methods to understand perceptions about their landraces. They use SSR markers for their genetic analysis. They found low genetic diversity within landraces compared to studies at regional scales, and significant differentiation among landraces suggesting barriers to gene flow probably due to differences in flowering time and variation in mating system among landraces. Farmers cultivate several landraces in the same field, thus the population size per landrace is small and the effect of drift, although variable, can be quite high. Due to the recognition and value given to different landraces farmers may preserve landrace identity by selecting panicles with specific characteristics for re-sowing. The authors found little evidence for spatial genetic structure and attributed this to widespread seed exchanges among farmers, suggesting that in spite of sorghum being mainly autogamous, there is substantial gene flow among landraces, underlying the important role of farmer practices in maintaining landraces identity and favoring genetic diversity.

**Sorghum in Kenya**

Laybeyrie et al. (2014) studied the association between sorghum spatial genetic patterns and ethnolinguistic differences in an area where three ethnic groups with different languages are in contact on the eastern slope of Mount Kenya, Kenya. The study was done within an area with minimal agro-ecological variation, ruling out this factor as a driver of the variation observed. Sorghum landraces were collected directly from farmers who were also interviewed. SSR markers were used to generate data for genetic analysis. The authors found that ethnic groups maintained different landraces, but that improved varieties were homogenously distributed across groups. Genetic differentiation among the landraces was not related to isolation-by-distance, but associated with the ethnic group to which farmers belonged. Genetic differentiation of sorghum landrace populations across ethnic groups was significant indicating that in a similar environment, ethnic differences and identities create boundaries that constraints seed flows and thus gene flow, with different seed selection criteria and practices across communities favoring morphological differentiation as well. The authors concluded that social relations and exchanges are important drivers of genetic structure in the landraces they studied.

**Barley in Ethiopia**

Samberg, Fishman, & Allendorf (2013) studied barley, an autogamous crop, in Ethiopia, collecting landraces, as well as interviewing farmers in 12 communities at different elevations in a North-South transect. They used morphological characteristics for their phenotypic analyses and SSR markers for their genetic analyses. They found that samples from low elevations were different to those at other elevations, showing particular distinctiveness and diversity. However, samples from other elevations that are more widely grown did not show structure by elevation, indicating that some seed exchange happens across altitudinal gradients for some varieties. They also found that six- and two-rowed barley types have distinct population structures. Two-rowed varieties showed spatial clustering between nearby communities and a good correspondence between the names given to them by farmers, physical characteristics and genetic structure. Six-rowed varieties, on the other hand, showed weak genetic structure and broad spatial patterns of variation along North-South and elevation gradients, suggesting high levels of seed exchange and thus gene flow across a broad spatial scale. The authors concluded that farmer management of seed and seed exchange leads to different sets of population processes in the same landscape: “isolation and diversification of low elevation varieties, broad geographic patterns in productive varieties and clustering by location and variety name in specialty varieties” (p. 12).

**Sorghum and pearl millet in Kenya**

Labeyrie et al. (2016) carried out a very interesting comparative study contrasting sorghum, an autogamous crop, with pearl millet, an allogamous one at ten sites occurring along two elevation transects and across six ethnolinguistic groups, on Mount Kenya. Studying these contrasting crops, allowed the authors to assess the effects of pollen-mediated versus seed-mediated gene flow, particularly since the latter is heavily influence by social and ecological processes. They collected samples of the landraces and improved varieties used, as well as conducted surveys with farmers. For genetic analyses they used SSR markers. In the case of pearl millet populations, the authors found an absence of genetic structure, with no genetic differences among sites, either due to high level of co-ancestry and/or genetic connectivity among them. The absence of genetic differences between local and foreign varieties seemed to be related to the strong effect of pollen-mediated gene flow. They attributed the observed patterns of genetic diversity to a combination of both high pollen-mediated gene flow among varieties and seed-mediated flow due to large diffusion and adoption of foreign varieties leading to genetic homogenization. On the other hand, in the case of sorghum populations they found genetic differentiation among sites, but the extent of this varied by altitude, being nil in the lowlands, but with significant differences at mid-altitude sites; genetic differentiation was also detected along the north to south transect. They attributed these geographic patterns to “the combination of limited pollen-mediated gene flow on a local scale and restricted seed-mediated gene flow among geographic areas” (p. 1242). Seed sourcing was similar for both crops—mostly being local and spatially confined to relatively small areas for landraces, but not for foreign varieties, which should constrained seed-mediated gene flow for the former, but not the latter. The study shows that pollen-mediated and seed-mediated gene flow have major but differential effects on diversity patterns depending on the crop breeding system, even with similar seed sourcing practices.

**Rice in China**

Wang et al. (2016) compared 24 pairs of landrace varieties of *Indica* (non-glutinous) and *Japonica* (glutinous) rice. The pairs were formed by matching samples collected around 1980 conserved in the National gene bank with a set collected by them on-farm directly from farmers, with the same name and from the same areas and environments. The recent samples were obtained from 12 villages of 9 different ethnic groups in three provinces of southwest China in 2014. Results show at least one statistically significant difference for at least one of four parameters related to genetic diversity and allelic polymorphism for nine of the 24 pairs. In all of these cases, parameter values were higher in 2014 than in 1980, and the authors conclude that for a significant number of landrace varieties genetic diversity had increased under on-farm conservation. In terms of specific alleles, results showed great variation among landraces collected at different times, with some alleles disappearing and new ones appearing. In 16 pairs there were more specific alleles in 2014 than in the 1980 populations. In general, alleles from landraces under on-farm conservation were richer than those under ex situ, suggesting that the former promotes the maintenance and development of genetic diversity. The genetic structure of landraces was significantly different between both types of conservation. The authors conclude that changes in genetic structure and diversity between landrace varieties conserved ex situ and on-farm requires the re-collection of landraces at regular intervals to insure the protection of their genetic diversity and integrity

**Potatoes in Peru**

Quiros et al. (1992) studied the diversity of potatoes in the Cusco region of Peru, collecting tubers from 18 markets and interviewing farmers about selection and management practices. The collected tubers were analyzed with isozymes. Cluster analysis of isozyme data showed the existence of four discrete major groups and six small heterogeneous groups. All tuber phenotypes were found in each of the major groups identified. Grouping was not associated with the market from which tubers were sampled. It appears that all genotypes belong to a single, large gene pool and that substantial gene flow between varieties of different groups may take place. In particular, this may be facilitated by markets where tubers from different locations are pooled together. It may seem surprising that gene flow occurs among crop populations that are vegetative propagated. The authors indicate that reproduction from true seed occurs from time to time, either as a purposeful farmer practice or inadvertently as a consequence of tuber selection of volunteer plants. The use of true seed generates segregation for skin and tuber colors and introgression of rare alleles, from which farmers may select desirable tubers, leading to the creating of new genotypes. The authors concluded that “Andean potato is a large and plastic gene pool being increased and renovated by outcrossing followed in some cases by human selection of desirable phenotypes” (p. 112).

de Haan et al. (2013) compared two samples of potatoes landraces from a highland region in central Peru, collected nearly 40 years apart, using SSR markers and morphological characteristics. One sample was obtained from the CIP gene bank and another from eight custodian farmers (knowledgeable farmers that maintain a high diversity of landrace varieties). Samples were selected so as to be able to compare a “spatially well-defined and representative ex situ population with a contemporary in situ population” (p. 508). Results show that both populations share a large proportion of alleles with individual SSR markers, but at the level of landrace cultivars, the in situ population includes most unique cultivars. There were no statistically significant differences between both populations in allelic diversity. Most variation was found within rather than among populations. However, the in situ population had a high presence of rare alleles (frequencies < 1%) indicating an uneven distribution pattern in farmers´ landrace stocks; while the ex situ population had a higher proportion of rare alleles (frequencies < 5%), suggesting a more even allele distribution. The facts that most alleles are shared between populations, but most unique alleles are either scarce (ex situ) or rare (in situ) suggest modest genetic change, or evolution with an influx of new and efflux of old alleles. For example, 12 unique alleles representing 8.5% of the total number were found exclusively in the in situ population, indicating evidence of the origination of new allelic diversity, “reaffirming the importance of in situ conservation to allow for seed-tuber flows and ongoing evolution.” (p. 515). At the same time, the detection of exclusive alleles ex situ supports the importance of conservation in gene banks. The authors conclude that their results support the complementary nature of ex situ and in situ conservation.

**Cassava in Guyana**

Elias et al. (2001) studied landrace varieties of cassava from one indigenous village in south-western Guyana. In this village, different varieties are mixed in the same field and farmers often exchange varieties within and among villages, usually with relatives in the latter case. Mostly varieties are vegetatively propagated from stem cuttings, but sometimes farmers incorporate into the propagation material volunteer cassavas grown from seeds (referred to as seedlings). The authors established a common garden experiment to compare phenotypic characteristics of the different varieties and hence assess morphological diversity, and used AFLP markers for genetic characterization. Results showed intra-varietal genetic variability and a high proportion of polymorphic varieties, in spite of strong bottlenecks due to the very low number of individuals that contribute genetically to the next generation. Farmers may incorporate seedlings into their repertoire of varieties either as a new variety or incorporate them into established varieties if the seedlings share morphological traits with them. Since there was a weak correlation between genetic composition and morphological structure, the incorporated seedlings are not expected to be genetically close, thereby introducing a new genotype into the established variety in which they are included, thereby adding further genetic diversity. These farmers favor diversification selection and retain varieties even if they are low yielding, thus maintaining a great amount of diversity. The farmers’ incorporation of seedlings from true seed, diversifying selection and the retention of low yielding varieties contribute to maintain a highly diverse set of cassava varieties, showing the important role that farmer practices play in the processes that maintain and generate crop diversity, even for vegetatively propagated crop.
